# The Story of the Insane from Year to Year

**Published:** 1895-01-12

**Authors:** 


### THE STORY OF THE INSANE FROM YEAR TO YEAR.

(7th Series.)

### DUNDEE ROYAL ASYLUM.

This report covers the period between June 20th, 1892, and June 19th, 1893. On the first-mentioned date the asylum contained 360 patients, and on the latter date the number had risen to 386. There were 188 admissions, 64 recoveries, and 42 deaths. The percentage of recoveries on admissions was 34·04, and the percentage of deaths on the average number resident was 11·26. Turning to the causes of insanity, we find hereditary influence is responsible for 58 of the year's admissions, intemperance in drink for 33, and domestic trouble for 14. Cerebral and spinal diseases caused death in 27 cases, and consumption in only one. Although the admissions are far in excess of the average of former years, Dr. Rorie does not think the increase is owing to an actual increase of insanity in the district, but, among other things, to the greater readiness of the public to send patients to the asylum, the old feelings of dislike and suspicion of asylums dying away, and to disinclination on the part of the friends of the insane to bear with them; and he further points out that there has been a widening of opinion on the part of the medical profession and the public as to the extent of insanity which justifies detention. No doubt these and other factors of asylum populations must be taken into account when we speak of the increase of the registered insane; but we fear that, after making full allowances, there is an increase of mental disease, although it would, perhaps, be difficult to prove either this or its opposite. The instruction of the nurses and attendants by lectures and special training has been continued, and seven of the staff have passed the examination of the Medico-Psychological Association. There are seventy-one private patients in the asylum, and these pay from £25 to £180 a year. The paupers are paid for at the rates of 11s. and 12s. 6d. per week.

### THREE COUNTIES' ASYLUM.

This is the asylum for Bedfordshire, Hertfordshire, and Huntingdonshire. On January 1st, 1893, it contained 1,030 patients, and on December 31st there were in residence 1,029. The admissions were 221, the recoveries 88, and the deaths 119. The percentage of recoveries on admissions is 39·8, and that of the deaths on the average number resident is 11·5. Hereditary influence and congenital defect account for 70 of the admissions, intemperance in drink for 15, and domestic troubles for 7. "Love" figures as the cause in 7, and religion in 4. Thirty-eight of the deaths were owing to cerebral and spinal diseases, and 18 to consumption. Dr. Swain contrasts the recovery rate in asylums of various sizes, and he finds that in those having over 2,000 beds it stands at 40·7; between 1,500 and 2,000 beds, at 38·4; between 1,000 and 1,500 beds, at 40·9; between 500 and 1,000 beds, at 38·3; and under 500, at 38·7. These figures, if they show anything at all, show that the size of an asylum has no great influence on the recovery rate, and that if an explanation of such difference be sought for, other causes must be explored. Dr. Swain, in a similar way, contrasts the weekly rate of maintenance, and finds that it is lowest in those asylums between 500 and 1,000 beds; but here, again, there must be many causes at work besides mere size. The asylum is about full, and the proposal to dissolve the union between the counties and build several asylums is the only perfectly satisfactory solution of the difficulty. The committee record "their approval of the zeal and ability with which Dr. Swain has conducted the important duties devolving on him." The weekly cost of maintenance per head is 8s. 7¾d.

